# Clinical evaluation study of the German network of disorders of sex development (DSD)/intersexuality: study design, description of the study population, and data quality

**DOI:** 10.1186/1471-2458-9-110

**Published:** 2009-04-21

**Authors:** Anke Lux, Siegfried Kropf, Eva Kleinemeier, Martina Jürgensen, Ute Thyen, The DSD Network Working Group

**Affiliations:** 1 Institute for Biometry and Medical Informatics, University Otto-von-Guericke, Leipziger Strasse 44, 39120 Magdeburg, Germany; 2 Department for Pediatric and Adolescent Medicine, University Clinic Schleswig-Holstein, Ratzeburger Allee 160, 23538 Lübeck, Germany

## Abstract

**Background:**

The German Network of Disorders of Sex Development (DSD)/Intersexuality carried out a large scale clinical evaluation study on quality of life, gender identity, treatment satisfaction, coping, and problems associated with diagnoses and therapies in individuals with disorders of sex development (DSD). DSD are a heterogeneous group of various genetic disorders of sex determination or sex differentiation, all of which are rare conditions. In about half of all cases the molecular genetic diagnosis is unknown and diagnosis rests on clinical features.

**Methods and design:**

The multi-centre clinical evaluation study includes short-term follow-up in some and cross-sectional assessments in all age and diagnostic groups fitting the criteria of DSD. Recruitment was from January 2005 until December 2007 in whole Germany and, additionally, in 2007 in Austria and German-speaking Switzerland. The study consists of a psychosocial inquiry for children, adolescents and their parents, and adults with standardized instruments and the collection of DSD-specific medical data by the attending physician. The main goal was the description of clinical outcomes and the health-care situation of individuals with DSD using a broad generic definition of DSD including all conditions with a mismatch of chromosomal, gonadal and phenotypical sex. 439 children and adolescents, their parents and adults with DSD participated.

**Discussion:**

The clinical evaluation study represents the most comprehensive study in this clinical field. The paper discusses the study protocol, the data management and data quality as well as the classification used, and it describes the study population. Given the lack of large datasets in rare conditions such as DSD and often biased results from small scale clinical case series, the study aims to generate concrete hypotheses for evidence-based guidelines, which should be tested in further studies.

## Background

Disorders of sex development (DSD) are a heterogeneous group of rare conditions. A German epidemiological study estimates a rate of 2.2/10 000 cases with ambiguous genitalia at birth at minimum [1]. Not included were conditions which are not obvious at birth but are diagnosed later in childhood or adolescence. Most specific conditions occur at a rate of 1:100.000 births or less. DSD are defined as congenital conditions with a mismatch of chromosomal, gonadal and phenotypical sex. A new nomenclature and classification has been suggested, delineating DSD with 46, XX karyotype and signs of androgen excess, namely congenital adrenal hyperplasia (CAH), and those with 46, XY karyotype and signs of complete or partial lack of virilization. The latter include partial and/or complete androgen insensitivity, complete and partial gonadal dysgenesis, and disorders of androgen hormone synthesis (e.g. 5α-reductase deficiency, 17β-hydroxysteroid-dehydrogenase deficiency). Sex chromosome anomalies such as Turner syndrome, Klinefelter-Syndrome, mixed gonadal dysgenesis, mosaics may be classified as DSD [2], but were not included in this study.

The rareness of a condition creates a serious challenge for research and patient care. Among approximately 30.000 diseases 5–7.000 are considered »rare« conditions with prevalence less than 1:2.000. Although rare, millions of people are affected worldwide but many lack access to specialized caregivers. The majority of conditions require extensive diagnostic procedures, ongoing symptomatic therapy and psychosocial support. Specific treatments often are not available and studies to promote evidence based treatment strategies are hampered by the heterogeneity of causes and clinical presentation, small numbers of individuals being treated in each centre, lack of incentives for the pharmaceutical companies to invest in research, and the lack of experience of the professionals involved. Therefore, the German Ministry of Education and Science (BMBF) has been funding ten disease-specific networks for rare diseases since 2003. Aim of this initiative is to promote national networks concerning research, specialized clinical and diagnostic centers and self-help and parent organizations.

One of the funded networks is the German Network DSD/Intersexuality, which started its work in October 2003. The network includes more than 40 institutions, 150 professionals and self-help groups in Germany for cooperation and will respond to the need for greater scientific knowledge in etiology and prognosis of various DSD conditions and improved health care. Overall goals were to develop guidelines for clinical care and support clinicians in coordination and collaboration, provide counseling, develop information and educational material for people with DSD, increase public knowledge about of the biology of sex development, and encourage respectful and appropriate communications with people affected. Central element of the network is the multi-centre clinical evaluation study. Part of the motivation for the research program was to develop appropriate methodologies to conduct clinical studies in rare conditions. Issues of heterogeneity of data, large variation among centers, small sample sizes in subgroups, age and gender appropriateness, validity of instruments, and interdisciplinary collaboration have to be addressed to be able to ensure the quality of data and develop analytical methods.

The aim of this paper is to describe and explain the study design, to characterize the study population, and to assess the data quality. We will describe specific critical issues for studies in the field of DSD in section 2. The special aims of the present study are presented in Section 3. Section 4 is directed to the study design and is followed by a specification of the data management. The study population is described in Section 6. In Section 7, the data quality is assessed. A discussion of the chances and limitations of the study completes the paper.

### 1. General problems of outcome studies in Disorders of Sex Development

Evidence-based treatment of patients with DSD is challenged by a dearth of clinical longitudinal outcome studies. Given the rarity of the condition, the heterogeneity of the clinical symptoms and variation in both surgical and medical treatments, evidence based guidelines are lacking beyond expert recommendations [3]. Therefore parents' and clinicians' decisions are often based on the clinicians' expertise, expert opinion and not on adequate data [4]. Reviews and studies about long-term outcomes concerning DSD mainly focus on psychosexual and surgical outcomes; e.g. [5-9]; especially in CAH; e.g. [10-25]. Obviously psychosexual aspects are important outcome parameters in context of DSD. Nevertheless, there are more criteria to evaluate treatment in individuals with DSD. There are few studies with such a broader spectrum of outcomes measured such as health related quality of life or mental health [26-36]. However in the majority of these studies, only special diagnostic groups are investigated and solely patients of a single medical institution or participants recruited by patient organizations have been inquired. Resulting problems are small sample sizes and recruitment biases. A more generic definition of DSD is important because condition specific studies will exclude people from research, as they do not fit the narrow categories (e.g. syndromal disorders, disorders of unknown etiology). Another problem is the missing comparability because every study employs other methods. Most of the DSD-studies could be classified as case series. Because of recruitment problems in context of rare disorders there are only poor quality cohort and case-control studies. Outcome studies concerning DSD are hampered by several problems (modified from Mazur) [7]:

#### 1. Lack of accurate diagnosis

The best choice is a molecular genetic characterization for inclusion criteria. However, in 50% of the cases the molecular diagnosis is missing. To integrate these patients in clinical outcome studies, strict clinical inclusion criteria are necessary.

#### 2. Selection bias

There is a selection bias when patients are recruited by former and actual care providers as well as by self-help groups. In many studies no information about the patients who refused to participate is available. Samples recruited by advocacy groups are not representative for the total population, because many patients are not organized in these groups. Patient registers with inclusion of cases at the time of diagnoses should greatly help to ensure proper representation, but they are only established in a few regions in Europe [37].

#### 3. Missing independence of care provider and researcher

Studies conducted by the clinical care provider may have increase responses of social desirability. Because of the sensibility of the nature of the subject, independent trained research teams should conduct these studies and assure that the personal information is not transferred to the care providers.

#### 4. Small sample sizes

One of the reasons is that many diagnoses are extremely rare. A broader recruitment strategy using strict clinical inclusion criteria such as the above mentioned generic DSD-definition [2] allows studies with larger samples.

#### 5. Lacking control groups

The described broad recruitment also opens the door for comparisons of different DSD-groups (e.g. girls/women with CAH vs. girls/women with partial androgen effects vs. girls/women with no androgen effects). Furthermore, patients with other chronic disorders and controls from the general public must be interviewed with the same battery of instruments where applicable.

#### 6. Lack of standardized instruments

There is a lack of standardized instruments to measure disease-specific quality of life and other subjective outcomes. Because of comparability, it seems necessary to establish batteries of instruments that will be used by different groups and to use the same descriptive terminology.

The described problems show that the rareness of disorders of sex development requires more national and international cooperation to increase sample sizes and to get high-quality outcome data. The German-wide outcome study was designed to overcome at least some of the described problems.

### 2. Overall purpose

The clinical evaluation study represents the most comprehensive study in this clinical field and includes short-term follow-up in some and cross-sectional assessments in all age and diagnostic groups. In preparation of the study, a large multi-professional research team discussed the inclusion of girls and women with CAH in a DSD-study. Many clinicians considered CAH as a metabolic disorder of the adrenal gland avoiding to address psychosexual issues in the care of their patients. However due to the ambiguous genitalia, medical, surgical and psychosocial problems may be similar as in patients with 46, XY karyotype and DSD; this opinion was confirmed by the inclusion of CAH in the classification of the consensus group later on [2]. The main outcomes in the study include the following parameters: phenotype of the external and internal genitalia, physical health including additional health problems, fertility, and sexual function in adults; social and psychosexual adjustment, mental health, quality of life, and social participation, social support, and coping. The inclusion of different etiological groups is important to generate hypotheses regarding the relevance of genetic, hormonal, psychological and social influence for the main outcomes. Typical treatment sequences and decisions for special interventions and treatment satisfaction are explored as potential co-factors. The main goal of the study is to generate concrete hypotheses for evidence-based guidelines, which should be tested in further studies. Until now, the scientific knowledge is not sufficient to generate a-priori hypotheses.

## Methods and design

### 1. Design, instruments, and logistics

#### Inclusion/exclusion criteria

Inclusion criteria of the study followed the above mentioned generic definition of DSD as a discrepancy of chromosomal, gonadal and phenotypical sex. We included both participants with a confirmed diagnosis of DSD (by molecular genetic testing, laboratory results, or histology) or a clinical diagnosis by a physician. Excluded were individuals with Klinefelter- or Ullrich-Turner-Syndrome, severe psychiatric comorbidity and mental disabilities. Included were all individuals meeting the mentioned criteria and living in Germany or German-speaking Switzerland or Austria.

#### Study design

The design was dependent on the allocation to subgroups related to age and time since first diagnosis:

a) Newborns, children and adolescents up to the age of 16 with a new diagnosis of a DSD within the last 6 months and their parents were contacted twice. After the baseline visit, there was a follow-up interview after 12 months (cohort).

b) Children and adolescents up to the age of 16 with a DSD prior 6 months before the study and their parents had only one interview (cross-sectional).

c) Adults with DSD had also only one interview (cross-sectional).

The sample size for this highly explorative study with a widespread field of outcome parameters in a heterogeneous population was primarily planned under the aspect of feasibility. Based on the available patients with DSD in Germany and on their assumed high interest in this study, we expected to include approximately 400 patients, which is much more than in previous studies in the literature. We concluded that the most relevant age groups would then cover about 80 to 120 patients, so that different subgroups regarding classification by diagnosis or treatment could attain a size of about 40. Such sample sizes enable to detect, e.g., differences of about 5 to 7 in the physical and mental sum score of the SF-36 (two-sided comparison of two groups, α = 0.05, standard deviation of about 10 and power of 80%), which is just a difference typical for some severe chronic diseases.

#### Instruments

There are few condition-specific instruments for DSD. We selected and developed different questionnaires after consulting experts in the field and extensive review of the literature. The Clinical Evaluation Study consists of two components:

1. *Psychosocial inquiry *for children, adolescents and their parents, and adults conducted by psychologists with standardized instruments during personal encounters: The battery of instruments for the psychosocial inquiry includes standardized and validated instruments in German language where available. Instruments suitable but not available in German have been translated. The translations were carried out according to international translation standards [38] with two separate backward and forward professional translations. Because of specific vulnerabilities in individuals with DSD, some items were modified slightly, e. g., replacing of the word "illness". The standardized and evaluated instruments are related to health-related quality of life, mental health, treatment satisfaction, coping, parenting stress, social support, body image, gender identity, and gender role behavior with different methods or specific formats for different age-groups including proxy-versions for parents. Specific condition related items were added in a self-constructed questionnaire. The self-constructed disease-specific DSD-questionnaires for parents, adolescents and adults cover major aspects concerning DSD and have been developed in cooperation with support groups and parent organizations (focus group discussions), and academic and medical experts. All DSD-questionnaires cover the domains sociodemographic information, child's or own social life, diagnostic procedures, medical and surgical interventions, exposure to DSD and experiences. Adults and parents are additionally asked about pregnancy and childbirth whereas adolescent's and adult's questionnaires contain items in context of partnership and sexuality. The adult DSD-questionnaire contains 112 items, the parent version 106 and the adolescent version 53 items. Children aged 4 or above participated in the study, answering some standardized questionnaires. An overview of the instruments used in the different age groups is given in Table 1. A more detailed description of all questionnaires is given in the additional file 1[39-58].

**Table 1:** Instruments used for different age groups. A detailed of the instruments is given in the additional file 1.

Instrument	Parents					Children		Adol.	Adults
Age (years)	0–0.5	0.5–3	4–7	8–12	13–16	4–7	8–12	13–16	>16

KINDL			X	X	X	X	X	X	
SF-36									X
SDQ			X	X	X			X	
BSI									X
CHC-SUN	X	X	X	X	X				
CSQ-8									X
CODI								X	
PSI		X	X	X	X				
FKSI						X	X		
BI-1								X	X
GII						X	X		
UGDS								X	
FGI									X
CBAQ			X	X					
GRQ						X	X		
Toy to keep						X	X		
DSD-parents	X	X	X	X	X				
DSD-adults									X
DSD-adolescents								X	
Medical data	answered by attending physician								

BI-1: Body Image Scale, BSI: Brief Symptom Inventory, CBAQ: Child Behavior and Attitudes Questionnaire, CHC-SUN: (Child Health Care – Satisfaction, Utilization and Needs, CODI: Coping questionnaire for children and adolescents, CSQ-8: Client satisfaction questionnaire, DSD: Disorder of Sex Development, FGI: Questionnaire of Gender Identity, FKSI: Frankfurt children's self-concept inventory, GRQ: Gender Role Questionnaire, GII: Gender Identity Interview, KINDL: Questionnaire for measuring health-related quality of life in children and adolescents, PSI: Parenting Stress Index, SDQ: Strengths and Health Questionnaire, SF-36: Health Survey, UGDS: Utrecht Gender Dysphoria Scale

2. The *Questionnaire "Medical Data" *was to be completed by the attending physician to document the medical history and diagnostic findings of each participant. The 64-item questionnaire is composed of four main thematic domains: pregnancy & delivery, diagnosis & initial findings (Prader stages, severity of hypospadias, external & internal genitalia, gonads, and genetics), therapeutic history (masculinizing & feminizing surgeries, surgical complications, and hormonal treatment), current/last findings (general health, external genitalia, Tanner stages, pubertal development, diagnosis). The development was assisted by a medical expert-group of network members. The questionnaire was adopted in a consensus meeting.

The study design, patient information and questionnaires battery were presented to and discussed with the European Scientific Advisory Board at a workshop at the ESPE meeting in Basel in September 2004.

The study project management and the biometry group met in May 2006 for an interim data analysis. All items of non-standardized questionnaires were checked for missing data and plausibility. Implausible items as well as such items having a very high missing rate have been removed from the non-standardized questionnaires in order to reduce the time effort and strain for the participants. One of the standardized instruments (coping in children) was dismissed as respondents apparently did not understand some of the questions and high rates of missing data.

#### Ethics

Ethics approval was sought first from the medical ethic committee at the University Lübeck. Subsequently ethic approvals were sought from the ethic review boards in the other study centers. An essential requirement was to develop and implement procedures to ensure data protection. Every participant received the same informed consent sheets, including information about data security and a waiver of consent to allow transmission of confidential medical information from the physician. All adolescents, patients and adults gave written consent to participation and release of medical data. Children were verbally asked to consent to participate after age-appropriate explanations. Parents could decline the participation of their children (4–12 years), but were allowed to participate themselves.

Policies and methods on data analysis and publication of results were discussed with co-investigators and partners of the clinical study. Network partners and researchers stating certain interests in subsets of the data may submit written proposals on data analysis including objectives, methods and anticipated results. After review, the scientific board will permit access to all or subsets of the data made anonymous, not permitting identification of personal data.

#### Recruitment

Recruitment started in December 2004 and interviews in January 2005. Because of their specialization in DSD, recruitment took place mainly in the study centers in Lübeck (north), Magdeburg/Berlin (east), Essen/Bochum (west) and Erlangen (south). Additionally, medical cooperation partners and self-help groups in Germany participated to encourage their patients/members to join the study and to dispense the information flyer. In Austria and Switzerland cooperation partners participated in St. Gallen, Bern, Vienna, Linz and Innsbruck (starting January, 2007). In the preparation phase, widespread information about the study was given to physicians via medical journals, scientific meetings, the network-DSD-homepage, personal communication and a newsletter from the DSD-network. We expected to recruit 400 participants between January 2005 and December 2007.

In case an individual was invited to participate in the study but declined, we collected some basic information (i.e. the recruiting study center and access to the study, age group, assigned gender, diagnosis and the time of first diagnosis of DSD), in order to perform a non-responder analysis at the end of the study.

#### Study logistics

Following the initial contact, a telephone intake call took place to explain the study, to explore the patient's interests and willingness to participate in any or all parts of the study and to arrange the realization of the interview. The study interviews took place at one of the study centers or at participant's home, depending on preferences of the participants. Data from individual patients have been collected as written questionnaires. Although information was collected as quantitative data in paper and pencil inquiries with only few open questions, the interviews always took place in personal attendance of one of the trained interviewers who were all psychologists. Due to the sensitive nature of the subjects and concerns about re-activation of past traumatic experiences, the European scientific advisory board of the study, had disapproved to send questionnaires per mail. An attending physician completed the questionnaire „medical data" with written consent by the participant. The study centre psychologist coded all study sheets according predefined coding instructions, removed personal data and created an individual identification number. After checking for missing and implausible data, staff sent the completed study sheets to Magdeburg for data entry.

### 2. Data management

The study data is kept in a Microsoft Access 2000 database consisting of several tables organized as groups of questionnaires. One of the tables was for the first-contact telephone call containing only data that were collected both for participants and non-participants. A second identical database was used for double entry. Both data bases are stored on the institute's server at the University of Magdeburg; routine backup was performed by the hospital's computing centre. In addition, a regular backup on an external disk kept separately in a secure place in another room was made. Access to the data base and to the backups was restricted to the staff directly included in the study. The server was not accessible via the web.

The data of each participant were entered into the database using Microsoft Access input forms. This included also a second line data check. In case of incomplete and obviously implausible data the data entry was suspended and a feedback was given to both the central study centre in Lübeck and the local study centers for further evaluation and correction. After completion of the queries data entry was continued. In order to ensure a high data quality, a second data entry was completed by another documentarian. In regular intervals the data bases for first and second data entry were checked and matched using the software SAS/PROC COMPARE.

For further statistical analysis, these tables were imported in the statistical software SPSS.

## Results

### 1. Description of the study population

#### General description

Overall 516 individuals were contacted for the study. Five of them had to be excluded due to applicable exclusion criteria – two with Turner syndrome, two with Klinefelter syndrome and one individual with mental retardation. 72 individuals denied participation. Thus a total of 439 participants were interviewed. This includes two children who died during the study. One child (newborn) died after the first visit and was lost for the follow-up visit, another child (age group 4–7 years) died before the interview, but the parents answered the DSD questionnaire.

Table 2 describes the study population with regard to the recruiting study centre, access to recruitment, the sex of rearing/recent gender, age groups, time of first diagnosis, and diagnosis related inclusion criteria (categories in latter are not mutually exclusive). Participants and non-participants are presented separately. Statistical analysis of the non-participants follows in the next section on data quality.

**Table 2:** Characteristics of study participants and eligible non-participants from the telephone intake call

	Recruited	Non-participation	Participation
	N	N (%)	N
Study Centers			
- Germany:			
North	130	17 (13%)	113
East	91	17 (19%)	74
South	119	16 (13%)	103
West	116	19 (16%)	97
- Switzerland	29	3 (10%)	26
- Austria	26	0 (0%)	26

First contact based on			
- Medical institutions/physician	439	61 (14%)	378
- Self-help groups	44	4 (9%)	40
- Internet	12	3 (25%)	9
- Other (e.g. friends, school, print media)	16	4 (25%)	12

Sex of rearing in children/recent gender in adults			
- Male	144	20 (14%)	124
- Female	367	52 (14%)	315

Age groups			
- Newborns	24	1 (4%)	23
- 6 months – 3 years	85	11 (13%)	74
- 4 – 7 years	84	4 (5%)	80
- 8 – 12 years	97	11 (11%)	86
- 13 – 16 years	80	14 (17%)	66
- Adults	141	31 (22%)	110

Time of the presumptive diagnosis			
- Prenatal	14	2 (14%)	12
- Perinatal	302	29 (10%)	273
- Postnatal ≤ 1 week	29	0 (0%)	29
- Later	117	12 (10%)	105
- Unknown	49	29 (59%)	20

Inclusion criteria			
- Gonadal dysgenesis	81	8 (10%)	73
- Disorders of androgen synthesis	21	2 (10%)	19
- Androgen insensitivity	60	8 (13%)	52
- Congenital adrenal hyperplasia (CAH)	202	28 (14%)	174
- Hypospadia	72	4 (6%)	68
- Other criteria (e.g. micropenis, complex malformation of the urogenital tract)	44	9 (20%)	35
- Suspected diagnosis of DSD	42	15 (36%)	27
- Unknown diagnosis of DSD	1	1 (100%)	0

Most active participants (378) were recruited via the study centers or approximately 60 cooperation partners of the study. Forty participants were recruited by self-help groups, 9 via internet. The proportion of self-referrals (i.e., recruited via self-help groups or internet) was larger in adult participants (23/110) than in parents (26/329). Of the 110 adult participants, 20 were active in self-help groups.

Altogether, 142 different physicians completed the questionnaire "medical data". Six of them completed more than ten and four of them more than 20 questionnaires. Most of the physicians (80,1%) completed only one or two questionnaires.

#### Classification of patients

The sample includes more than 30 different diagnoses as stated in the "medical data" questionnaire. Twelve individuals have an unclassified diagnosis of DSD. 54 individuals with the clinical diagnosis "severe hypospadia" with unknown underlying disorder participated. According to the treating physician, in 342 participants (77.9%; excluding the 54 patients with hypospadia) the final diagnosis has been confirmed by genetic, laboratory, endoscopic or other tests.

Most of these diagnoses occurred in very small numbers limiting power for statistical analyses. To be able to group patients, we decided to use a classification by karyotype, signs of androgen effects (XY-DSD and XX-DSD as suggested by the consensus statement of the ESPE/LWSPE conference) and sex of rearing/recent gender (9 adults stated to live neither in female nor male gender, in one child there is no definite decision about sex of rearing. Because of statistical reasons, they were integrated into the classification system. The child was integrated in the DSD-XY-P-F group because of her own declaration of being a girl. The decision for the 9 adults was based on the official status in the civil registries.), which combines the most important aspects regarding physical condition, effects of socialization and experiences of individuals with DSD [32]. Sex of rearing is used for children and adolescents on the basis of the parent's decision whereas the term recent gender role is used for adolescents.

##### Subgroup 1 (DSD-XX-P-F)

Individuals with XX-karyotype and partial androgen effects, reared as girls/living as women (n = 178). This subgroup consists in great part of women with CAH, but includes also few individuals with aromatase deficiency.

##### Subgroup 2 (DSD-XX-P-M)

Individuals with XX-karyotype and partial androgen effects, reared as boys/living as males (n = 9). This includes the diagnoses CAH, true hermaphroditims and XX-male (SRY-translocation). Males with XX-karyotype and partly androgen effects and the diagnosis of true hermaphroditism and SRY-translocation were reassigned to subgroup DSD-XY-P-M, because we presumed the presence of some material of the "Y"-chromosome. The XX-males (n = 2) with CAH are removed from statistical analysis due to small numbers, where group comparisons were conducted.

##### Subgroup 3 (DSD-XX-C-F)

Individuals with XX-karyotype and without androgen effects, reared as girls/living as women (n = 3). The group contains individuals with agonadism, ovarian failure and complete gonadal dysgenesis. It is debatable to include women with XX-karyotype and ovarian failure or complete gonadal dysgenesis in the group of DSD. We did so a priori, because they lack typical male or female sex development. However, due to small sample size this group was excluded from statistical analysis on a group level.

##### Subgroup 4 (DSD-XY-P-F)

Individuals with XY-karyotype and partial androgen effects, reared as girls/living as women (n = 96). Included are the diagnoses pAIS, true hermaphroditism, gonadal dysgenesis, 17β-HSD, 5alpha-RD, micropenis, severe hypospadia and individuals with a clinical diagnosis of DSD fitting the criteria. *Subgroup 5 (DSD-XY-C-F): *Individuals with XY-karyotype and without androgen effects, reared as girls/living as women (n = 39). The group includes individuals with cAIS, complete gonadal insufficiency and Swyer-Syndrome.

##### Subgroup 6 (DSD-XY-P-M)

Individuals with XY-karyotype and partial androgen effects, reared as boys/living as men (n = 114). Included are patients with pAIS, true hermaphroditism, gonadal dysgenesis, 17β-HSD, 5alpha-RD, micropenis, hypospadia and individuals with a clinical diagnosis of DSD fitting the criteria.

For statistical group comparisons we used four subgroups (1, 4, 5, and 6- the latter including few individuals from subgroup 2) in the following analyses. Table 3 gives an overview for these groups including the definition and the composition with respect to clinical diagnoses and to age groups. There is evidence that high concentrations of pre- and postnatal androgens contribute to the development of male-typical behavior, whereas low levels of androgens will induce female-typical behavior [6,59,60]. Therefore it seems important to compare children with DSD with complete lack and those with partial lack of androgen effects. In addition, this grouping takes effects of gender-typical socialization and potential effects of karyotype into account. Nevertheless, this discussion is not finished yet. The above classification will be primarily used to test hypothesis on the influence on diagnoses and related treatments on all the outcome variables considered in the study, not least to reduce the multiplicity of hypotheses even though all future analyses of these data will be exploratory. In secondary analyses, alternative classifications will be considered in special problems and then we shall investigate which classification is best related to the outcome of interest. First analyses, however, deflate the expectation to these analyses (e.g., classification and regression trees or cluster analyses) because the different classification systems are related to each other and in any case there will a large unexplained proportion.

**Table 3:** Definition and composition of primary classification groups for the Clinical Evaluation Study

Group	1. DSD-XX	2. DSD-XY-P-F	3. DSD-XY-P-M	4. DSD-XY-C-F
Definition:	Girls/women with 46, XX DSD and signs of over-virilization	Girls/women with 46, XY DSD and partial signs of virilization	Boys/men with 46, XY DSD and signs of under-virilization	Girls/women with 46-XY DSD without any signs of virilization
Karyotype	XX	XY^+^	XY^+^	XY^+^
Sex of rearing/recent gender	Female	Female	Male	Female
Androgen effects	present	present	present	none

Diagnoses contained	CAH (172) True hermaphroditism (1) partial gonadal dysgenesis (2) aromatase deficiency (3)	pAIS (18) gonadal dysgenesis (partial, mixed) (43) disorder of androgen biosynthesis (21) true hermaphroditism (4) associated malformations with DSD (cloacal extrophy) (2) penis agenesis (2) severe hypospadia (1) unclassified clinical diagnosis of DSD (5)	severe hypospadia (53) gonadal dysgenesis (mixed, partial) (25) pAIS (12) disorders of AMH (1) disorders of androgen biosynthesis (10) true hermaphroditism (2) associated malformations with DSD (cloacal extrophy) (1) micropenis (3) disorder of androgen syndromal disease (1) epispadias (1) XX-male* (3) unclassified clinical diagnosis of DSD (9)	cAIS (23) compl. gonadal dysgenesis (14) gonadal dysgenesis with complete female phenotype and 45, X/46XY (1) unclassified clinical diagnosis of DSD (1)

Age distribution:				
Infants < 6 months	8	4	10	1
6 months – 3 years	29	7	36	1
4 – 7 years	29	12	34	5
8 – 12 years	33	24	21	8
13 – 16 years	33	19	7	7
Adults	46	30	13	17

Total n	178	96	121	39

^+ ^incl. mosaics with parts of „Y chromosome" (in some cases chromosome status has not been investigated, in these cases classification results according the clinical status)

* despite 46, XX karyotype

Excluded from group comparisons:

46, XX; CAH & male sex of rearing, n = 2

46, XX & without androgen effects (e.g. gonadal insuffiency), n = 3

### 2. Assessment of data quality

In order to assess the quality of the data, different aspects will be considered here: selection bias, heterogeneity, missing questionnaires, missing items, accordance between different questionnaires and outliers in the data. All statistic analyses reported below were performed with the statistical software package SPSS, version 15. Tests with p-values below 0.05 were considered as significant. Due to the study design, all analyses are exploratory.

#### Selection bias

The selection bias was studied by comparing the individuals who declined participation with those who participated stratified by certain characteristics (study centre, access to the study, sex of rearing, age group, time of the first diagnosis of DSD).

When using the chi-squared test for bivariate analysis, there were no significant differences between participation and refusal in relation to study centre, access to the study and sex of rearing on bivariate analysis (cf. Table 2). The comparison of the age groups showed a higher rates of non-participants in adults (p = 0.002). Regarding the time of first diagnosis of DSD, there were only an increased proportion of non-participants in those patients with unknown time of first diagnosis. Type of diagnosis did not result in different rejection rates with exception of individuals with severe hypospadia who declined participation less frequently (p < .05). When checking these results for mutual confounding of the different factors in a logistic regression with all factors considered simultaneously, then the effect for hypospadia was no longer significant, indicating confounding effects of age here.

#### Homo-/heterogeneity between study centers

The different study centers (North, East, South and West Germany, Switzerland and Austria) were compared in chi-squared tests for possible heterogeneity of recruited patients with respect to socio-demographic characteristics as nationality, religion, size of home town, educational level and employment (of parents and adults only).

The distribution of age groups and sex of rearing was similar across study centers. The proportion of participants with a mirgational background was about 10% in all centers. As expected, there were differences between German study centers in terms of religion (p = 0.004 and 0.001, respectively, with a smaller proportion of religious people in the eastern part), reflecting the composition of the corresponding populations. Similarly, a larger percentage of adult patients was out of job (p = 0.028) and more mothers were working in a full time job (p = 0.002) in the eastern part of Germany. In Austria and Switzerland, there were smaller proportions of participants living in towns with more than 100,000 inhabitants than in Germany (p < 0.001) and we found different educational levels of the mothers in the three countries (0.040). Also between the four German centers differences have been found in the educational level with a higher level in the eastern part for parents of DSD children (p = 0.003 and 0.037, respectively) but a larger proportion of college graduates in the northern and western part for the adult patients (p = 0.039). As both parents and adults should be of similar age, that can no longer be explained by differences in the reference population and it could be an indication of different living conditions of DSD patients in the former eastern and western part of Germany.

#### Questionnaire and item non-response for standard instruments

In participants, we calculated the proportion of totally missing questionnaires and of items not completed on each standardized instrument. Problems with incomplete response occurred particularly in children and adolescents, particularly because of missing parental consent or of tiredness, and we searched in this subgroup for possible influences on the completion or rejection of single questionnaires and for non-response in single items.

Table 4 gives an overview on the percentages of missing response to standardized instruments resulting in loss of items, of entire sub-scale (which can sometimes be calculated despite single missing items) and of whole questionnaires.

**Table 4:** Percentage of missing of entire questionnaires, single items and scores in standardized instrument^#^.

	% non-response to entire questionnaires	% missing items in questionnaires completed	% missing sub-scale scores
Children/adolescents			
- KINDL	10.8	0.8	1.0
- SDQ	9.1	0.0	0.0
- CODI	10.6	0.4	0.3
- FKSI	12.1	1.3	0.7
- GII	18.2	4.0	*
- UGDS	9.1	2.4	*
- GRQ	10.9	3.2	*
- SS-A	10.6	2.1	0.0
- BI-I	9.1	2.2	0.5

Parents			
- KINDL	1.3	1.8	1.8
- SDQ	1.7	0.1	0.1
- CBAQ	2.4	10.3	2.9
- PSI	0.0	0.6	0.8
- CHC-SUN	0.3	4.9	7.3

Adults			
- SF-36	0.0	0.4	0.1
- BSI	0.0	0.1	0.0
- FGI	0.0	1.4	1.2
- SS-A	0.0	0.7	0.0
- CSQ-8	0.0	0.8	*
- BI-I	0.0	2.8	2.7

BI-1: Body Image Scale, BSI: Brief Symptom Inventory, CBAQ: Child Behavior and Attitudes Questionnaire, CHC-SUN: (Child Health Care – Satisfaction, Utilization and Needs, CODI: Coping questionnaire for children and adolescents, CSQ-8: Client satisfaction questionnaire, DSD: Disorder of Sex Development, FGI: Questionnaire of Gender Identity, FKSI: Frankfurt children's self-concept inventory, GRQ: Gender Role Questionnaire, GII: Gender Identity Interview, KINDL: Questionnaire for measuring health-related quality of life in children and adolescents, PSI: Parenting Stress Index, SDQ: Strengths and Health Questionnaire, SF-36: Health Survey, UGDS: Utrecht Gender Dysphoria Scale

* = not applicable

# The percentages for items and for subscale scores are averaged over all items or subscale scores of the questionnaire. The calculations for these two columns do not include those persons who omitted the entire questionnaire (cf. second column).

In children/adolescents the non-response rate to entire questionnaires ranged from 9.1% for the SDQ and UGDS to 18.2% for the GII. In parents this rate is always below 2.5% (highest for CBAQ with 2.4%) and no questionnaires are missing in adult participants.

On the average children/adolescents failed to complete 1.7% of items in questionnaires they completed. For questionnaires administered to parents, the proportion of items not completed ranged from 0.1% for SDQ to 10.8% for CBAQ. In adults, the average proportion of items not completed is 0.6%.

The proportion of missing sub-scale scores on questionnaires completed by children varied from 0% for SDQ to 1% for KINDL. For parents, the proportion of missing scores ranged from 0.1% for SDQ to 2.9% for CBAQ, and for questionnaires administered to adults, the proportion of missing subscale scores varied from 0% for BSI to 1.2% for FGI.

Overall, the proportions of completely filled questionnaires calculated in relation to all participants, were satisfactory in most of the questionnaires: in children and adolescents 193 of 232 (83.2%) answered the KINDL, 120 of 166 (72.3%) the FKSI, 60 of 66 (90.9%) the SDQ (self-report adolescents only), 53 of 66 (80.3%) the CODI (adolescents only). However, response rates were insufficient in some of the questionnaires: 94 of 166 (56.6%) answered the GII, 92 of 166 (55.4%) the GRQ, and 43 of 66 (65.1%) the UGDS, (adolescents only).

In order to detect possible influences of the age groups, gender and diagnosis (in the sense of the above classification with four groups) in the subgroup of children and adolescents, we carried out bivariate comparisons (chi-squared tests) for each of these factors and multivariate comparisons (logistic regression) for mutual adjusted comparisons.

With respect to the percentage of completely missing questionnaires, only the age group showed significant influences (both in the unadjusted and adjusted comparisons) for the KINDL, the FKSI, the GII and the GRQ, where the younger group (age 4 – 7) hat the largest proportion if missing questionnaires. Related to the number of missing items in the available questionnaires, there was only a significant effect of age for the GII, where the younger group (age 4–7) had less missings (both adjusted and unadjusted). An influence of the diagnosis was seen in the unadjusted test for UGDS (with largest percentage of missings for DSD-XY-P-F) but the significance disappeared in the adjusted comparison, so that it could be an indirect effect of gender and age.

#### Completeness of medical data

The medical data questionnaire answered by a medical doctor was available for all participants. The self constructed DSD questionnaire was answered by the parents and by the adult participants in all cases, six adolescents (9%) did not respond to this questionnaire. Table 5 gives an overview of missing items as average over groups of items.

**Table 5:** Percentage of missing items in DSD-questionnaires, averaged over groups of items

Topic	% missing in DSD quest. for adolescents	% missing in DSD quest. for parents	% missing in DSD quest. for adults	% missing in medical data questionnaire
Person, family, social contacts	5.0	2.5	0.7	*
Pregnancy, birth	*	0.3	1.0	1.3
Development of child	*	5.4	*	*
Diagnostics	9.1	3.8	2.4	4.2
Treatments	4.2	6.7	5.4	5.2
Experiences	5.5	5.4	0.5	*
Sexuality	7.8	*	10.0	*
Actual findings	*	*	*	10.3

*' = not applicable

In adolescents, some questions showed poorer response rates than others. The highest average proportion of missing values was 9.1% in the area of diagnostic procedures and 7.7% in the area of sexuality. In the parent DSD-questionnaires, most missing values concerned treatment (6.7%), and in the adult DSD-questionnaires, questions about sexuality had the highest rate of missing values (10%).

In the medical questionnaire, we found missing values in some areas – the proportion varied between 1.3% for questions about pregnancy and birth and 10% for questions about actual findings.

#### Discrepancies between the self-administered DSD questionnaires and the medical questionnaire

Some items were recorded both in the medical questionnaire (filled in by the physicians) and in the DSD questionnaires (completed by the patients or their parents). The consistency of related items from different instruments is investigated in cross-classifications of the corresponding items.

A comparison of the answers showed substantial differences in items related to current height and weight, data on pregnancy and birth, data on suspected diagnosis and diagnosis, data on karyotype and data on surgical treatments and hormone therapy. A selection of deviating data is represented in Table 6.

**Table 6:** Percentage of participants with disagreements between physicians reported medical data and self-response in DSD questionnaires

Compared items	Disagreement	
	Number	%
Current height (adolescents/adults, deviation > 5 cm)	3	1.8
Weight (adolescents/adults, deviation > 5 kg	8	4.7
Mode of delivery	9	2.0
Medication during pregnancy	43	9.8
Week of gestation at birth	87	19.8
Length at birth, deviation > 4 cm	8	1.8
Birth weight, deviation > 400 g	6	1.4
Age at the time of presumptive diagnosis		
- deviation ≥ 3 months	43	9.8
- deviation ≥ 6 months	34	7.8
- deviation ≥ 12 months	19	4.4
- deviation ≥ 24 months	13	3.0
- deviation ≥ 48 months	7	1.6
Signs and symptoms leading to the suspected diagnosis of DSD (varying for different symptoms)	2 – 89	0.5 – 20.3
Karyotype	36	8.2
Current diagnosis	48	10.9
Number of operations (by adolescents)	13	21.0
Surgical treatment	15	3.4
Type of the surgical treatment (varying for treatments)	3 – 67	0.7 – 15.3
Previous hormone therapy	96	21.9
Current hormone therapy	35	7.8

#### Outliers in psychological outcome

Another aspect of data quality in the study is the occurrence of outliers, here particularly considered in the psychological outcomes. We checked outliers in all sub-scales both in the total study population and in subgroups for age and for diagnosis using an outlier definition as conventional for box plot presentations: an outlier is a value which is more than 1.5 inter-quartile range above/below the upper/lower quartile of that variable.

The rates are similar across age groups and also across diagnosis groups (not shown here in detail). There is no age group or diagnosis group with a relevant increased percentage of outliers. The proportions vary slightly among different psychological constructs. Table 7 presents the number (as range over the different subscales of an instrument) and percentage (averaged over all sub-scales) of outliers separately for children/adolescents, parents and adult patients.

**Table 7:** Number and percentage of outliers in standardized instruments

Questionnaire	Number of outliers in sub-scales from – to	% (average over all sub-scales)
Children/Adolescents		
- KINDL	0 – 9	1.4
- SDQ	0 – 4	2.1
- CODI	0 – 1	0.3
- FKSI	0 – 2	0.5
- GII	9 – 17	7.9
- UGDS	2	3.0
- GRQ	0	0.0

Parents		
- KINDL	2 – 11	2.6
- SDQ	0 – 4	1.1
- CBAQ	1 – 2	0.9
- PSI	0 – 17	2.1

Adults		
- SF-36	0 – 25	4.8
- BSI	0 – 4	0.7
- FGI	0 – 11	7.1

BI-1: Body Image Scale, BSI: Brief Symptom Inventory, CBAQ: Child Behavior and Attitudes Questionnaire, CHC-SUN: (Child Health Care – Satisfaction, Utilization and Needs, CODI: Coping questionnaire for children and adolescents, CSQ-8: Client satisfaction questionnaire, DSD: Disorder of Sex Development, FGI: Questionnaire of Gender Identity, FKSI: Frankfurt children's self-concept inventory, GRQ: Gender Role Questionnaire, GII: Gender Identity Interview, KINDL: Questionnaire for measuring health-related quality of life in children and adolescents, PSI: Parenting Stress Index, SDQ: Strengths and Health Questionnaire, SF-36: Health Survey, UGDS: Utrecht Gender Dysphoria Scale

A check of the individual data showed that outliers in different outcomes are generally not caused by the same individuals. For example, among 44 children and adolescents with outliers in anyone instrument only seven had outliers in two different questionnaires. Among 68 parents with outliers, only ten had outliers in two questionnaires and five in three different questionnaires; among 49 adults with outliers, six had outliers in two different questionnaires and two in three different questionnaires. Also outliers in more than sub-scale of an instrument were rare.

## Discussion

The Clinical Evaluation Study of the German DSD network presented here is the largest and most comprehensive study on quality of life, psychosexual development, treatment satisfaction, coping, and related problems in persons with DSD available at present. In a re-iterative consensus process the study group has made important decisions about ways to classify individuals in clinically appropriate groups to allow analysis on a group level. The Consensus statement of the ESPE/LWSPE was instrumental in this process. We decided to combine the principle of a generic classification based on karyotype and type of genetic disorder with the fact in which gender role (female/male) the individual is being raised (in children) and actually lives. Alternative proposals included:

1. Classification by gender, arguing with gender as a social construction. We felt that such a classification would combine individuals with very different hormone exposure and treatment experiences, i.e. those with CAH and cAIS, both living as women. However, for some aspects of analysis, for example differences in social participation, researchers may use such a simple grouping in certain circumstances.

2. Classification by phenotype of genitalia (defined e.g. according to Prader classification). The underlying hypothesis of this approach is that the degree of genital virilization may be used as an indicator for prenatal androgen effects on brain development [20,61-63]. There are several reasons why we decided not to follow the grouping according to Prader-stage: The first and most important reason is, that there is no scientific evidence for a direct relationship between the degree of genital virilization and the masculinization of the brain [64,65]. Another reason arises from our poor data quality on Prader-stage, both at birth/time of diagnosis (17.1% unknown or missing) and currently (20.0% unknown or missing). Data quality is limited not only because of missing data, but also because of the retrospective collection of data, coming from patient's records rarely completed by DSD-specialists. As a result, we found some implausible data such as a Prader-stage 3 in individuals with cAIS.

Therefore, we chose the above classification in 4 subgroups. We acknowledge the fact that the classification may be suitable for our purposes with a focus on psychosocial outcomes such as psychosexual development, social participation, psychological well-being and health related quality of life. A large body of literature on the impact of chronic conditions in childhood has shown that the psychosocial aspects of patient care, family adaptation, coping strategies and social support are of greater importance than the individual diagnosis [66]. This does by no means challenge other classification systems that may be better suited for example for treatment outcomes, biological studies on pathophysiology, or genetic studies.

The classification scheme combining the karyotype, androgen effects and actual gender role has certain advantages: (1) it allows inclusion of individuals with extremely rare conditions, so that their information can be analyzed within a meaningful comparison of individuals with similar conditions; (2) it allows individuals with tentative diagnoses to participate if they met the generic inclusion criteria for DSD; (3) it takes into consideration the effects of androgens on the developing brain which are thought to affect gender-typical behavior and gender identity; and (4) it includes social aspects of living with a chronic condition.

Although the study has been well prepared in several steps assuring an adequate study design, appropriate instruments, implementing standard operating procedures and safeguarding data protection, several limitations have to be noted.

### Representativeness

The study may be not completely representative for individuals with DSD. We attempted to evaluate possible selection bias by recording some baseline data of non-participants and found only a few characteristics overrepresented in those who had declined participation, indicating only mild effects of but not excluding selection bias. We explicitly found an influence of age with adult persons being less willed to participate. Presumably, their commitment to the attending physicians inviting them to participate in the study is less pronounced compared to younger age-groups.

Other factors not reflected in our data come from the psychologists conducting the interviews. They encountered several problems in recruiting participants for the study:

(1) Some physicians taking care of individuals with DSD did not wish to cooperate with the network at all, particularly in clinics for patients with CAH. In an inquiry in 2005 among medical cooperation partners within the network, the clinicians stated, that they would not inform approximately 10% of their patients. Specific reasons included: 1. no disclosure about the diagnosis, 2. unwillingness to accept a given diagnosis as a form of "DSD/Intersexuality", or 3. concerns about the psychological impact caused by the interviews.

(2) Another problem of recruitment emerged in respect to newborns with DSD age 0 to 6 months and individuals with a new diagnosis of a DSD within the last 6 months. These two groups turned out to be extremely burdened by the diagnostic process and coping with a DSD-diagnosis, and were not open to participate in a study. Thus just the two cohorts with intended follow-up are very small (23 newborns and 3 patients with a new diagnosis, responded to the follow-up interview), so that the longitudinal part of the study focused on changes in the outcome cannot seriously be analyzed.

Generally, we cannot exclude that persons with particularly poor experience in their medical history are less presented in the study. Nevertheless, we hope that the broad inclusion of patients from medical centers, and self-identified members of self-help groups and the internet reduced the bias. Even the latter groups had to agree to consent to release a physician from the professional secrecy and collection of medical data. This excludes individuals not in contact with the medical system.

### Homogeneity/heterogeneity of study centers

As described above, the study centers are homogeneous with respect to age, gender and nationality of their patients. The observed heterogeneity regarding religion and unemployment just reflects the corresponding differences in the population in general, and is not specific for persons with DSD or their families. That might be not the case for the educational level with different distributions for adult patients and the parents of children with DSD which could be a hint for specific conditions in different regions.

Thus, particularly analyses for quality of live, which is often related to the educational level, and with respect to access and satisfaction with patient care, should be conducted including the study centre as a co-factor in order to avoid a potential confounding for certain socio-demographic and health care related characteristics.

### Missing response to entire questionnaires or to items and subscales

The rates of non-response to entire questionnaires is generally low, indicating that the opportunity to answer the questionnaires with an interviewer present and to ask questions was successful to keep missing items as small as possible. The problem was most prominent in children, because of missings due to lack of parental consent for some or all children questionnaires. The percentage of missing items is acceptable – also because of the technical interim check of instruments combined with some reduction of problematic items. The remaining problems in some single items are due to the very intimate nature of the study and were hardly to prevent.

### Consistency of medical data

Another limitation of the study – related to the fact that it is a purely observational study – is that the medical data coming from physicians on the basis of their medical records may be not up-to-date at the time of the request to answer the questionnaire. That explains, e.g., the disagreement in the data for body height or body weight between questionnaires answered by patients and those from the physicians. Furthermore, the large proportion of physicians (about 80%) with only one or two patients included in the study indicates that at least some of them are not very experienced in the field of DSD, which may have negative effects on the data quality. Indeed, more detailed analyses, presented here, revealed also conflicts between the diagnoses stated and the accompanying information for a few patients. The only resort from this problem would have been the integration of a medical examination and possibly further testing by experienced endocrinologists. Aside from the enormous costs for such a study, we were anxious that this may have prevented even more individuals and families to participate due to the sensitive nature of the condition. The study design and recruitment allowed us to recruit the planned number of persons with DSD. The parallel questioning of attending physicians and the individuals (or parents) assured at least some degree of validation of the diagnoses and treatment related data. The remaining uncertainty has to be addressed in the ongoing main analyses of the study by sensitivity analyses with different choices for suspect diagnoses.

Thus summarizing, the study has good chances to reveal new insight in the quality of life, gender identity, treatment satisfaction and further related outcome in persons with DSD and in its relevant impact factors. This will be the concern of future publications based on the present paper. The problems mentioned are to some degree unavoidable in studies in such a highly sensitive field. For the reasons discussed and with a careful interpretation of the results, they should not stress the credibility of the study results.

## Abbreviations

BI-1: Body Image Scale; BSI: Brief Symptom Inventory; CAH: Congenital adrenal hyperplasia; CBAQ: Child Behavior and Attitudes Questionnaire; CHC-SUN: Child Health Care – Satisfaction Utilization and Needs; CODI: Coping questionnaire for children and adolescents; CSQ-8: Client satisfaction questionnaire; DSD: Disorder of Sex Development; FGI: Questionnaire of Gender Identity; FKSI: Frankfurt children's self-concept inventory; GRQ: Gender Role Questionnaire; GII: Gender Identity Interview; KINDL: Questionnaire for measuring health-related quality of life in children and adolescents; PSI: Parenting Stress Index; SDQ: Strengths and Health Questionnaire; SF-36: Health Survey; UGDS: Utrecht Gender Dysphoria Scale.

## Competing interests

The authors declare that they have no competing interests.

## Authors' contributions

All authors were involved in the development and implementation of the study from the beginning. AL and SK constructed the data base and were responsible for data management. EK and MJ coordinated the study as project managers and carried out one third of the interviews. UT supervised the study as principal investigator. Within this paper AL and SK were responsible for the statistical analysis and EK, MJ and UT for the medical and psychosocial aspects. All authors read and approved the final manuscript.

## Pre-publication history

The pre-publication history for this paper can be accessed here:



## Supplementary Material

Additional file 1 **Instruments used in the Clinical Evaluation Study**. The data provide an overview and detailed information about the instruments used in the Clinical Evaluation study, including target group, subscales, modifications, and specifications. Click here for file
