# Supplementary material for: Clinical evaluation study of the German network of disorders of sex development (DSD)/intersexuality: study design, description of the study population, and data quality
Source: BMC Public Health. 2009 Apr 21;9:110. doi: 10.1186/1471-2458-9-110 (PMC2678119; doi:10.1186/1471-2458-9-110)
Supplement: Additional file 1 — Instruments used in the Clinical Evaluation Study. The data provide an overview and detailed information about the instruments used in the Clinical Evaluation study, including target group, subscales, modifications, and specifications. [file 1471-2458-9-110-S1.doc]

Additional file 1: Instruments used in the Clinical Evaluation Study

| **Name of Instrument** | **Target Group** | **Refe-rences** | **Sub-scales** | **Specifications & modifications by the study group** |
| --- | --- | --- | --- | --- |
| Health-Related Quality of Life | | | | |
| KINDL^R^ Questionnaire for measuring health-related quality of life in children and adolescents | Self-assessment of children and adolescents (4-16 years)  Proxy-versions for parents of children and adolescents (4-16 years) | [39] | 6 sub-scales: physical, emotional, self-esteem, family, friends, school |  |
| SF-36 Health Survey - German version | Self-assessment: 14 years and older | [40] | 8 sub-scales: physical function, role physical, bodily pain, general health, vitality, social function, role emotional and mental health | → participants 17 year and older |
| Mental Health | | | | |
| Strengths and Health Questionnaire (SDQ) - German version | Self-assessment: 11-16 years  Proxy-versions for parents: (3-4 years, 4-16 years) | [41,42] | 5 sub-scales: emotional symptoms, conduct problems, hyperactivity/inattention, peer relationship problems, pro-social behavior | → self assessment for participants 13-16 years  → parents of children 4-16 years (proxy-version) |
| Brief Symptom Inventory (BSI) - German version | Self-assessment: 13 years and older | [43] | 9 sub-scales: somatization, obsessive-compulsive, interpersonal sensitivity, depression, anxiety, hostility, phobic anxiety, paranoid ideation, psychoticism | → participants 17 years and older |
| Treatment Satisfaction | | | | |
| CHC-SUN – Child Health Care – Module 2 (Satisfaction with care) & general satisfaction with care | Questionnaire for parents of children with chronic illness | [44] | 6 sub-scales: Diagnosis, coordination, child-centered care, hospital/clinical environment, doctor’s behavior, school, 1 additional item: general satisfaction | →because of the special situation of shame & privacy in context of DSD, we do not apply the school services scale  →we replaced the word “illness” by “disorders of sex development”  → all participating parents  → integrated in DSD-questionnaire for parents |
| CSQ-8  The client satisfaction questionnaire – German version | Self-assessment: 16 years and older | [45,46] | One-factor solution | →the word “illness” has been exchanged by the word “specialty of sex development” |
| Coping | | | | |
| CODI Coping questionnaire for children and adolescents | Self-assessment: 8-16 years | [47] | 6 coping-strategies: emotional reaction, cognitive-palliative, acceptance, distance, wishful thinking, avoidance | →the word “illness” was eliminated and the adolescents decided which word best fits their disorder  → self assessment for participants 13-16 years |
| Parental Impact | | | | |
| Parenting Stress Index (PSI)  German version | Parent self-report | [48,49] | 13sub-scales:  Parent domain: competence, social isolation, attachment to child, health, role restriction, depression, spouse  Child domain: distractibility/hyperactivity, adaptability, reinforces parent, demandingness, mood, acceptability  Additional scale: social support | → all participating parents of children older than 6 months |
| Social Support | | | | |
| The Social Support Appraisal Scale (SS-A)  German version | Self-assessment: 12 years and older | [50,51] | 5 sub-dimensions: perceived social support by friends, perceived social support by the family, network satisfaction  additional: perceived social support by the partner | → participants 13 years and older  → integrated in DSD-questionnaire for adolescents and adults |
| Self-concept & Body Image | | | | |
| Frankfurt children’s self-concept inventory (FKSI) | Self assessment: 3-12 years | [52] | 11 Sub-scales: physical appearance , physical sensation, physical efficiency, emotional disposition, anxiety, [self](http://dict.leo.org/?p=2Ib6..&search=self) [assurance](http://dict.leo.org/?p=2Ib6..&search=assurance), morality, cognitive efficiency, [self-assertion](http://dict.leo.org/?p=2Ib6..&search=self-assertion) and [assertiveness](http://dict.leo.org/?p=2Ib6..&search=assertiveness), interpersonal skills | → participants 4-12 years  → appliance of 5 sub-scales: physical appearance , physical sensation, physical efficiency, [self](http://dict.leo.org/?p=2Ib6..&search=self) [assurance](http://dict.leo.org/?p=2Ib6..&search=assurance), [self-assertion](http://dict.leo.org/?p=2Ib6..&search=self-assertion) and [assertiveness](http://dict.leo.org/?p=2Ib6..&search=assertiveness)  → development of 6 additional items concerning children’s satisfaction with body parts and 2 items concerning shame about physical appearance. |
| Body Image Scale (BI-1 | Self-assessment 12 years and older | [53] | 3 scales: primary gendaral characteristics, secondary gendaral characteristics, hormonally unresponsive characteristics | → participants 13 years and older  → integrated in DSD-questionnaire for adults and in the DSD-questionnaire for adolescents  → German translation |
| Gender Identity | | | | |
| Gender Identity Interview for Children | Self assessment: 4-12 years (girl’s and boy’s version) | [54] | 2 Scales: Affective gender confusion, cognitive gender confusion | → German translation |
| Utrecht Gender Dysphoric Scale | Self-assessment: 12 years and older | [55] | One-factor solution | → participants 13 years and older  → German translation |
| Questionnaire of Gender Identity | Self-assessment: 17 years and older | [56] | 4 Sub-Scales: feminine gender identity, masculine gender identity, transgender-scale, scale on certainty to belong to one specific gender |  |
| Gender Role Behaviour | | | | |
| Child Behaviour and Attitudes Questionnaire for boy and girls (CBAQ) Short-form | Proxy-version for parents (4-12 years) | [57] | 4 Sub-scales: femininity scale, cross-gender scale boys and girls, cross-gender scale boys, cross-gender scale girls | → German translation |
| Toy to keep | Self assessment: 4-12 years | [58] | One-factor solution | → toy sample for German children |
| Gender Role Questionnaire | Self-assessment: 3-12 years | unpubl. | One-factor solution | → participants between 4 &12 years  → German translation |
| Questionnaires concerning Intersexuality | | | | |
| DSD-Questionnaire for parents | Parents for children with DSD | developed with the study | Thematic domains: sociodemographic information, pregnancy and childbirth, child’s social life, child’s development, questions concerning DSD, medical intervention and surgery associated with the DSD, exposure to DSD and experiences | → newly developed, max.106 items |
| DSD-Questionnaire for adults | Adults (17 years and older with DSD) | developed with the study | Thematic domains: sociodemographic information, pregnancy and childbirth, social life, questions concerning DSD, medical intervention and surgery associated with the DSD, sexuality and partnership, exposure to DSD and experiences | → newly developed, max. 112 items |
| DSD-Questionnaire for adolescents | Adolescents (13-16) with DSD | developed with the study | Thematic domains: sociodemographic information, friends, sexuality, knowledge concerning DSD, medical intervention and surgery associated with the DSD, exposure to DSD and experiences | → newly developed, max. 53 items |
| Questionnaire “Medical Data” | Attending physicians | developed with the study | Thematic domains: pregnancy & course of labour, diagnosis and initial findings (external &internal genitalia, gonads, genetics), patient’s therapeutic history (surgeries, surgical complications, hormonal treatment), current / last findings (general health, external genitalia, puberty, diagnosis) | → newly developed, max. 64 items |
